# Cabozantinib versus placebo in patients with radioiodine-refractory differentiated thyroid cancer after prior vascular endothelial growth factor receptor-targeted therapy (COSMIC-311): outcomes by BRAF status

**DOI:** 10.3389/fonc.2026.1748566

**Published:** 2026-03-04

**Authors:** Marcia S. Brose, Bhumsuk Keam, Jolanta Krajewska, Ana O. Hoff, Fernanda Vaisman, Chia-Chi Lin, Erika Hitre, Daniel W. Bowles, Bruce Robinson, Steven I. Sherman, Nuttapong Ngamphaiboon, Xiang Guo, Andrew Simmons, Denise Williamson, Svetlana Andrianova, Nicholas Berry, Jaume Capdevila

**Affiliations:** 1 Department of Medical Oncology, Sidney Kimmel Comprehensive Cancer Center, Jefferson University, Philadelphia, PA, United States; 2 Department of Internal Medicine, Seoul National University Hospital, Seoul, Republic of Korea; 3 Department of Nuclear Medicine and Endocrine Oncology, Maria Skłodowska-Curie National Research Institute of Oncology Gliwice Branch, Gliwice, Poland; 4 Department of Endocrinology, Instituto do Cancer do Estado de São Paulo, Universidade de São Paulo, São Paulo, Brazil; 5 Department of Endocrinology, Instituto Nacional de Câncer, Rio de Janeiro, Brazil; 6 Department of Oncology, National Taiwan University Hospital, Taipei, Taiwan; 7 Department of Medical Oncology, The Multidisciplinary Head and Neck Cancer Center, Országos Onkológiai Intézet, Budapest, Hungary; 8 Division of Medical Oncology, Department of Medicine, University of Colorado Anschutz Medical Campus, Aurora, CO, United States; 9 Department of Medicine, Royal North Shore Hospital, University of Sydney, Sydney, NSW, Australia; 10 Department of Endocrine Neoplasia and Hormonal Disorders, University of Texas MD Anderson Cancer Center, Houston, TX, United States; 11 Department of Medicine, Ramathibodi Hospital, Mahidol University, Bangkok, Thailand; 12 Department of Translational Medicine and Bioinformatics, Exelixis, Inc., Alameda, CA, United States; 13 Department of Biostatistics, Exelixis, Inc., Alameda, CA, United States; 14 Department of Late Phase Clinical Development, Exelixis, Inc., Alameda, CA, United States; 15 Department of Medical Affairs, Exelixis, Inc., Alameda, CA, United States; 16 Department of Medical Oncology, Vall d’Hebron University Hospital, Vall d’Hebron Institute of Oncology (VHIO), IOB Quiron-Teknon, Barcelona, Spain

**Keywords:** *BRAF*, *BRAF* ^V600E^, cabozantinib, COSMIC-311, PFS, RAIR-DTC, TKI

## Abstract

**Background:**

Cabozantinib is approved for previously treated radioiodine-refractory differentiated thyroid cancer (RAIR-DTC) based on improved progression-free survival (PFS) versus placebo in the COSMIC-311 study. The *BRAF*^V600E^ mutation is common in DTC and is associated with poor prognosis. This planned exploratory analysis of COSMIC-311 reports outcomes by BRAF status.

**Methods:**

In this exploratory analysis, outcomes by *BRAF*^wt^ (wild-type) or *BRAF*^V600E^ status were evaluated in the COSMIC-311 phase 3 study in patients with RAIR-DTC who had previously received lenvatinib and/or sorafenib.

**Results:**

BRAF status was available for 106 of 258 patients enrolled in COSMIC-311; of these, 74 had *BRAF*^wt^ and 27 had *BRAF*^V600E^. Cabozantinib prolonged PFS versus placebo in both the *BRAF*^wt^ (hazard ratio [HR] 0.23 [95% CI: 0.12–0.44]; median PFS, 11.1 versus 1.9 months) and *BRAF*^V600E^ (HR 0.15 [95% CI: 0.04–0.59]; median PFS, 9.2 versus 1.9 months) subgroups. While no responses were observed with placebo in both *BRAF* subgroups, objective response rates (ORRs) of 11% and 18% were observed with cabozantinib in the *BRAF*^wt^ and *BRAF*^V600E^subgroups, respectively. Among patients treated with cabozantinib, 68% of the *BRAF*^wt^ group and 53% of the *BRAF*^V600E^ group reported grade 3/4 treatment-emergent adverse events; the incidences were 17% and 50% in the corresponding groups treated with placebo.

**Conclusions:**

In this subgroup analysis of COSMIC-311, cabozantinib improved PFS and ORR versus placebo irrespective of *BRAF* mutation status. Thus, cabozantinib is an efficacious treatment option with a manageable safety profile for previously treated patients with RAIR-DTC, including those with *BRAF*^V600E^.

## Introduction

The prognosis for patients with differentiated thyroid cancer (DTC) is generally favorable (1). Treatment options include active surveillance, surgery, and radioiodine (RAI) therapy. However, up to 15% of patients can develop RAI-refractory DTC (RAIR-DTC) that has less favorable outcomes and requires more aggressive management (2, 3). Genetic mutations or rearrangements as well as signaling pathway dysregulation are shown to drive the evolution of RAIR-DTC (3). For example, activating mutations of B-type raf kinase (*BRAF*), neurotrophin receptor tyrosine kinase (*NTRK*), or receptor tyrosine kinase rearranged during transfection (*RET*), have been associated with poor prognosis in patients (3).

First-line treatment options for RAIR-DTC, in the absence of *NTRK* or *RET* fusion, are the vascular endothelial growth factor receptor (VEGFR)-targeted tyrosine kinase inhibitors (TKIs) lenvatinib or sorafenib (4, 5). However, most patients eventually develop treatment resistance and experience disease progression. Until recently, no treatment options were available for patients experiencing disease progression after VEGFR-targeted therapy (6).

Cabozantinib, a multitargeted TKI, has been approved for previously treated patients with RAIR-DTC (7). In the United States, cabozantinib is indicated for patients aged ≥12 years who progressed after VEGFR-targeted therapy (7). In the EU, it is approved for adults who progressed after systemic therapy (8). These approvals were based on the findings of the phase 3 COSMIC-311 clinical trial (NCT03690388) in which cabozantinib significantly prolonged progression-free survival (PFS) and increased objective response rate (ORR) compared with placebo, with a manageable safety profile, in previously treated patients with RAIR-DTC (6). This clinical benefit was maintained during the extended follow-up period (median, 10.1 months) with no new safety signals (9, 10). Cabozantinib inhibits tyrosine kinases VEGFR, AXL, and MET, which are known to mediate tumor growth and angiogenesis in DTC (11). MET and AXL also promote resistance to VEGFR-pathway inhibition (12–14). Thus, the observed clinical benefits of cabozantinib in DTC previously treated with VEGFR-targeted therapies such as lenvatinib or sorafenib may result from cabozantinib targeting pathways associated with resistance.

The *BRAF*^V600E^ pathogenic variant is a constitutively active form of the BRAF kinase that drives disease evolution in multiple tumor types including DTC (15). Although there are other *BRAF* mutations, *BRAF*^V600E^ is the most common, occurring in 30–90% of DTCs, with the higher prevalences occurring in papillary or papillary-follicular histological subtypes (16–20). *BRAF*^V600E^ is the most common genetic change in RAIR-DTC, where it drives dedifferentiation and impairs expression and trafficking of proteins needed for iodine uptake (3, 15). *BRAF*^V600E^ is associated with aggressive tumor phenotypes, reduced response to RAI, and worse prognosis in RAIR-DTC (16–21).

Cabozantinib is approved for patients with RAIR-DTC whose disease has progressed after prior VEGFR-targeted therapy (22). As the *BRAF*^V600E^ mutation is prevalent and pathogenically important in RAIR-DTC, we explored the impact of BRAF status on cabozantinib outcomes in RAIR-DTC in a planned exploratory analysis of COSMIC-311 trial data.

## Methods

Design, ethics, eligibility criteria, stratification, randomization, treatment, assessments, and outcome measures of the COSMIC-311 randomized, double-blind, placebo-controlled phase 3 trial have been published previously (6). In brief, patients with RAIR-DTC ≥16 years of age previously treated with lenvatinib or sorafenib, never exposed to selective BRAF small-molecule inhibitors, and experiencing disease progression were randomized 2:1 to receive oral cabozantinib 60 mg/day or placebo. A blinded independent radiology committee (BIRC) adjudicated ORR and PFS as co-primary endpoints and determined best percent reduction in target lesion size according to Response Evaluation Criteria in Solid Tumors (RECIST) v1.1 as in the primary publication (6, 23). Other endpoints included disease control rate (DCR), the total proportion of patients who achieved a confirmed complete or partial response or stable disease (SD) at any time, and disease stabilization rate (DSR), the proportion of patients achieving a confirmed complete response, partial response, or SD lasting ≥16 weeks (6).

Tumor biopsy at the time of enrollment was optional. Tumor tissue (fresh or archival) from the most recently collected sample prior to enrollment was obtained for biomarker analysis. BRAF status was determined centrally for patients with available tissue via whole exome sequencing from formalin-fixed paraffin-embedded tumor tissue and paired blood samples. Somatic variants were determined using the variant caller VarDict (24).

The current exploratory subgroup analysis evaluated ORR and PFS in patients randomized to cabozantinib versus placebo by *BRAF*^wt^ (wild-type) or *BRAF*^V600E^ status. Best reduction in target lesion size per BIRC was visualized as waterfall plots. Kaplan-Meier curves were drawn for PFS and median PFS times estimated using the Kaplan-Meier method, hazard ratio (HR) for BIRC-adjudicated PFS was determined by a Cox proportional hazards model, and BIRC-adjudicated ORR was compared using an unstratified two-sided Fisher’s exact test (6).

## Results

Among 258 patients randomized into the COSMIC-311 trial, BRAF status was available for 106. Of these, 74 were *BRAF*^wt^ and 27 had the *BRAF*^V600E^ pathogenic variant. Of the 74 patients with *BRAF*^wt^, 44 were in the cabozantinib arm and 30 were in the placebo arm, and of the 27 patients with *BRAF*^V600E^, 17 were in the cabozantinib arm and 10 were in the placebo arm. Patients with *BRAF*^wt^ had papillary or follicular histology; papillary tumor histology was universal among patients with *BRAF*^V600E^ (**Table 1**). Baseline demographic and clinical characteristics are shown in **Table 1** and were consistent with those previously reported for the overall population of the COSMIC-311 trial (9).

**Table 1:** Baseline demographics and clinical characteristics.

Characteristic	*BRAF* ^V600E^		*BRAF* ^wt^	
	Cabozantinib (n = 17)	Placebo (n = 10)	Cabozantinib (n = 44)	Placebo (n = 30)
Age, median (range), y	70.0 (54–82)	62.0 (47–78)	66.0 (31–79)	65.5 (51–79)
≥65 y, n (%)	12 (71)	4 (40)	25 (57)	17 (57)
Female, n (%)	11 (65)	5 (50)	27 (61)	15 (50)
Race, n (%)				
White	12 (71)	8 (80)	41 (93)	21 (70)
Asian	3 (18)	2 (20)	2 (5)	5 (17)
Black	0	0	0	1 (3)
Other/unknown	2 (12)	0	1 (2)	3 (10)
Geographic region, n (%)				
Europe	8 (47)	2 (20)	28 (64)	15 (50)
Asia	2 (12)	2 (20)	1 (2)	4 (13)
United States/Canada	3 (18)	2 (20)	4 (9)	8 (27)
Rest of the world	4 (24)	4 (40)	11 (25)	3 (10)
ECOG performance status, n (%)				
0	8 (47)	6 (60)	21 (48)	15 (50)
1	9 (53)	4 (40)	23 (52)	15 (50)
Histological subtype, n (%)				
Papillary	17 (100)	10 (100)	17 (39)	14 (47)
Follicular	0	0	27 (61)	16 (53)
Number of previous VEGFR-TKIs, n (%)				
1	14 (82)	9 (90)	32 (73)	20 (67)
2	3 (18)	1 (10)	12 (27)	10 (33)
Metastatic lesions, n (%)^a^				
Bone	6 (35)	3 (30)	23 (52)	14 (47)
Liver	2 (12)	0	13 (30)	3 (10)
Lungs	13 (76)	9 (90)	35 (80)	20 (67)
Other	17 (100)	7 (70)	36 (82)	27 (90)

a Per investigator; patients may have had multiple lesions.

*BRAF*, B-type raf kinase; ECOG, Eastern Cooperative Oncology Group; TKI, tyrosine kinase inhibitor; VEGFR, vascular endothelial growth factor receptor; wt, wild type.

Clinical responses to cabozantinib were observed irrespective of patients’ BRAF status (**Table 2**). The ORR was 11% (95% CI: 3.8%–24.6%) and 18% (95% CI: 3.8%–43.4%) in the *BRAF*^wt^ and *BRAF*^V600E^ groups, respectively; all were partial responses. Patients randomized to placebo did not exhibit any responses irrespective of BRAF status. DCR and DSR were higher with cabozantinib than placebo irrespective of BRAF status. The DCR observed with cabozantinib was 75% in the *BRAF*^wt^ group and 88% in the *BRAF*^V600E^ group, contrasting with placebo DCRs of 33% in the *BRAF*^wt^ group and 30% in the *BRAF*^V600E^ group. DSR in patients in the cabozantinib arm were 52% and 65% in the *BRAF*^wt^ and *BRAF*^V600E^ groups, respectively, compared with 20% and 10% in the corresponding groups in the placebo arm.

**Table 2:** Tumor response per RECIST v1.1 by BIRC.

Tumor response	*BRAF* ^V600E^		*BRAF* ^wt^	
	Cabozantinib (n = 17)	Placebo (n = 10)	Cabozantinib (n = 44)	Placebo (n = 30)
ORR, % (95% CI)	18 (3.8–43.4)	0 (0.0–30.8)	11 (3.8–24.6)	0 (0.0–11.6)
Best overall response, n (%)				
Complete response	0	0	0	0
Partial response	3 (18)	0	5 (11)	0
Stable disease	12 (71)	3 (30)	28 (64)	10 (33)
≥16 wk	8 (47)	1 (10)	18 (41)	6 (20)
Progressive disease	1 (6)	6 (60)	5 (11)	19 (63)
No measurable disease	0	0	1 (2)	0
Missing/not evaluable	1 (6)	1 (10)	5 (11)	1 (3)
Median duration of response (95% CI), months	10.22 (NE–NE)	NE	NE (9.33–NE)	NE
DCR (CR+PR+SD), % (95% CI)	88 (63.6–98.5)	30 (6.7–65.2)	75 (59.7–86.8)	33 (17.3–52.8)
DSR (CR+PR+SD ≥16 wk), % (95% CI)	65 (38.3–85.8)	10 (0.3–44.5)	52 (36.7–67.5)	20 (7.7–38.6)

BIRC, blinded independent radiology committee; *BRAF*, B-type raf kinase; CR, complete response; DCR, disease control rate; DSR, disease stabilization rate; NE, not evaluable; ORR, objective response rate; PR, partial response; RECIST, Response Evaluation Criteria in Solid Tumors; SD, stable disease; wt, wild type.

**Figure 1:**
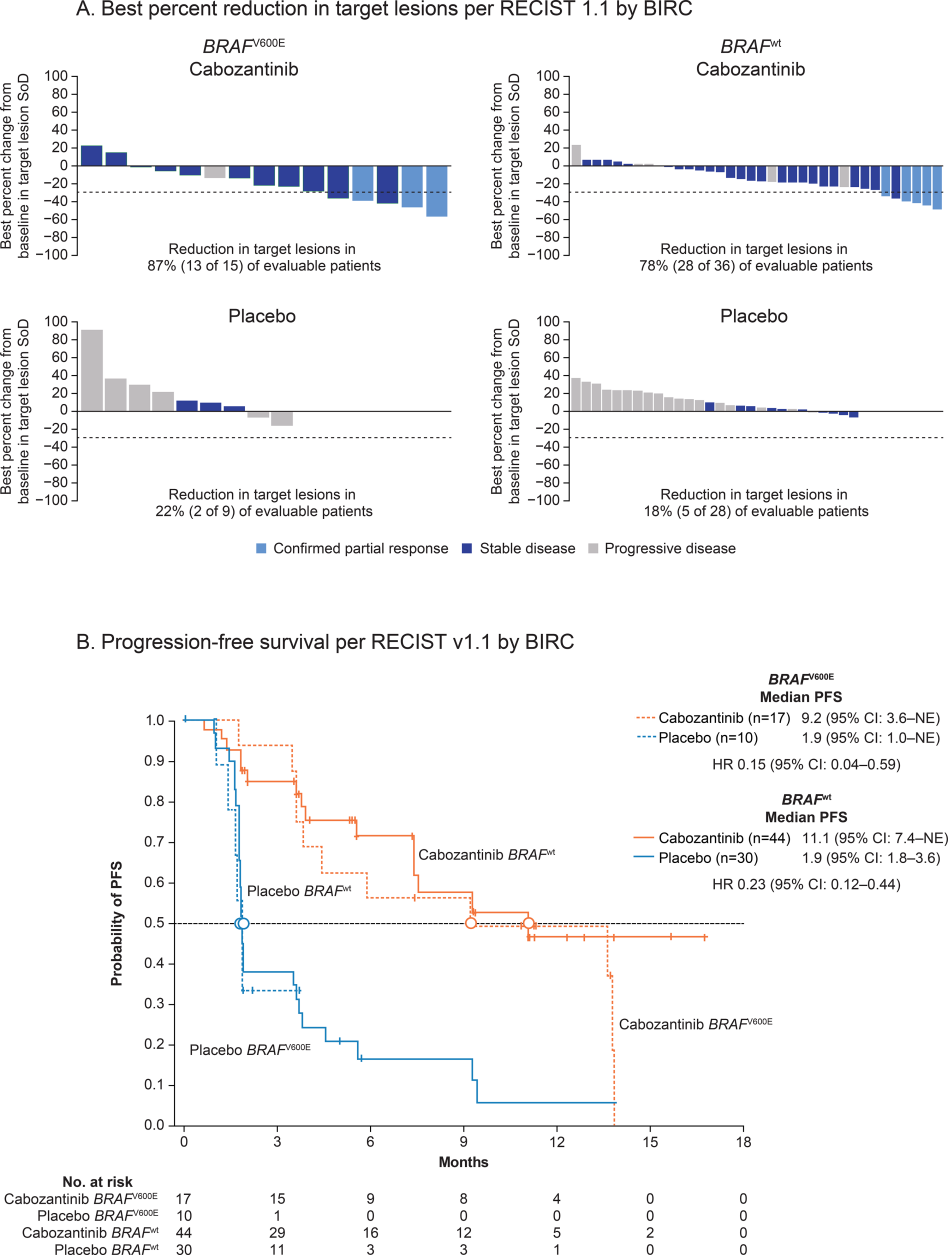
**(A)** Best percent reduction in target lesions per RECIST v1.1 by BIRC. Included only evaluable patients who had baseline and at least one post-baseline tumor assessment. **(B)** Progression-free survival per RECIST v1.1 by BIRC. BIRC, blinded independent radiology committee; *BRAF*, B-type raf kinase; CI, confidence interval; HR, hazard ratio; NE, not estimable; PFS, progression-free survival; RECIST, Response Evaluation Criteria in Solid Tumors; SoD, sum of diameters; wt, wild type.

Cabozantinib also reduced target lesions in a higher percentage of evaluable patients than placebo irrespective of BRAF status. In the cabozantinib arm, 78% and 87% of patients with *BRAF*^wt^ and *BRAF*^V600E^, respectively, exhibited target lesion reductions compared with 18% and 22% in the corresponding groups in the placebo arm (**Figure 1A**).

Cabozantinib was associated with prolonged PFS versus placebo irrespective of BRAF status. Among patients with *BRAF*^wt^, median PFS was 11.1 months in the cabozantinib arm versus 1.9 months in the placebo arm (HR 0.23 [95% CI: 0.12–0.44]). Among patients with *BRAF*^V600E^, median PFS was 9.2 months in the cabozantinib arm versus 1.9 months in patients in the placebo arm (HR 0.15 [95% CI: 0.04–0.59]; **Figure 1B**).

The safety profile of cabozantinib in both BRAF status groups was consistent with that seen in earlier reports of the overall population (6, 9). Among patients treated with cabozantinib, 68% of the *BRAF*^wt^ group and 53% of the *BRAF*^V600E^ group reported grade 3/4 treatment-emergent adverse events (TEAEs); the incidences were 17% and 50% in the corresponding groups treated with placebo. The most common grade 3/4 TEAEs in patients treated with cabozantinib (*BRAF*^wt^ and *BRAF*^V600E^ subgroups, respectively) were palmar-plantar erythrodysesthesia (9% and 24%), hypertension (11% and 18%), and hypocalcemia (11% and 18%) (**Table 3**). The most common grade 3/4 TEAEs in corresponding groups of patients treated with placebo were hypocalcemia (0% and 20%), hypertension (3% and 10%), and increased alanine aminotransferase (0% and 10%).

**Table 3:** Treatment-emergent adverse events.

Event	*BRAF* ^V600E^				*BRAF* ^wt^			
	Cabozantinib (n = 17)		Placebo (n = 10)		Cabozantinib (n = 44)		Placebo (n = 30)	
	Any Gr	Gr 3/4	Any Gr	Gr 3/4	Any Gr	Gr 3/4	Any Gr	Gr 3/4
Any event, n (%)	17 (100)	9 (53)	9 (90)	5 (50)	44 (100)	30 (68)	26 (87)	5 (17)
Diarrhea	9 (53)	2 (12)	0	0	32 (73)	1 (2)	2 (7)	0
Palmar-plantar erythrodysesthesia	10 (59)	4 (24)	0	0	20 (45)	4 (9)	0	0
ALT increased	9 (53)	1 (6)	1 (10)	1 (10)	11 (25)	0	0	0
AST increased	9 (53)	0	0	0	9 (20)	0	1 (3)	0
Decreased appetite	5 (29)	1 (6)	1 (10)	0	13 (30)	0	5 (17)	0
Hypertension	9 (53)	3 (18)	1 (10)	1 (10)	11 (25)	5 (11)	1 (3)	1 (3)
Hypocalcemia	6 (35)	3 (18)	2 (20)	2 (20)	16 (36)	5 (11)	0	0
Weight decreased	4 (24)	0	0	0	11 (25)	2 (4)	1 (3)	0
Nausea	2 (12)	0	0	0	19 (43)	1 (2)	1 (3)	0
Stomatitis	1 (6)	0	0	0	6 (14)	1 (2)	0	0
Asthenia	3 (18)	1 (6)	0	0	7 (16)	1 (2)	5 (17)	0
Fatigue	9 (53)	0	0	0	15 (34)	5 (11)	4 (13)	0
Mucosal inflammation	3 (18)	0	0	0	7 (16)	1 (2)	0	0
Vomiting	3 (18)	0	0	0	7 (16)	1 (2)	4 (13)	0
Hypomagnesemia	3 (18)	0	0	0	9 (20)	0	0	0
Proteinuria	5 (29)	0	0	0	6 (14)	3 (7)	1 (3)	0

Adverse events occurring in ≥15% of either treatment arm of the overall study population. ALT, alanine aminotransferase; AST, aspartate aminotransferase; *BRAF*, B-type raf kinase; Gr, grade; wt, wild type.

## Discussion

In this planned exploratory subgroup analysis of COSMIC-311, treatment with cabozantinib improved efficacy outcomes versus placebo irrespective of BRAF status. The ORR in the cabozantinib arm was 11% in the *BRAF*^wt^ group and 18% in the *BRAF*^V600E^ group, contrasting with 0% in both groups in the placebo arm, with substantially higher DCR and DSR among patients treated with cabozantinib. Cabozantinib prolonged median PFS to 11.1 months in the *BRAF*^wt^ group and 9.2 months in the *BRAF*^V600E^ group, contrasting with 1.9 months in both corresponding groups assigned to placebo. Thus, the co-primary outcomes of COSMIC-311 showed efficacy across *BRAF* subgroups at a comparable magnitude to the full study population (ORR: cabozantinib 11%, placebo 0%; median PFS: 11.0 months with cabozantinib, 1.9 months with placebo) (9). These findings are particularly important since the *BRAF*^V600E^ variant has been associated with poor prognosis, tumor aggressiveness, persistence, recurrence, and short survival in DTC (3).

A combination of dabrafenib (BRAF inhibitor) and trametinib (MEK inhibitor) currently has a tumor agnostic approval in the United States for patients with *BRAF*^V600E^ mutations, including anaplastic thyroid cancer but not specifically for RAIR-DTC, based on efficacy in the phase 2 ROAR and NCI-MATCH basket studies (25–27). The first prospective clinical trials with vemurafenib or dabrafenib provided evidence of activity of BRAF inhibitors in patients with RAIR-DTC and *BRAF* pathogenic variants (28, 29). Objective responses were generally higher in TKI-naïve patients compared with previously TKI-treated patients, but comparisons are limited due to small sample size and the variability and number of prior therapies. A more recent phase 2 trial of dabrafenib or dabrafenib plus trametinib in *BRAF*-mutated RAIR-DTC confirmed similar activity between single-agent BRAF therapy and combination BRAF plus MEK therapy in predominantly TKI-naïve patients (30). A global phase 3 study is ongoing to evaluate dabrafenib in combination with trametinib versus placebo in TKI-refractory *BRAF*^V600E^ RAIR-DTC (NCT04940052), which may provide evidence for utilizing *BRAF* and MEK*-*targeted therapy in TKI-exhausted RAIR-DTC patients. While these studies provide evidence of activity for the BRAF plus MEK inhibitor combination in *BRAF*-altered RAIR-DTC, the safety and efficacy of BRAF-targeted therapies have not been directly compared to TKIs (25–27). In the absence of randomized trials comparing BRAF-targeted agents to VEGFR-TKIs in *BRAF*^V600E^ RAIR-DTC, relative safety and efficacy of these agents, as well as the optimal sequencing of these treatment options remains unknown. To this end, a randomized phase 3 ECOG-ACRIN study EA3231 (NCT06475989) has been initiated to compare cabozantinib versus dabrafenib plus trametinib in patients with *BRAF*^V600E^ RAIR-DTC who have progressed on one or two prior VEGFR-TKI therapies. Results of this study will provide insight into the relative benefit of maintaining VEGFR inhibition after progressing on initial VEGFR-TKI therapy versus switching to BRAF and MEK inhibition. Further studies are needed to inform the appropriate sequencing of TKI and BRAF plus MEK inhibitors, with the goal of maximizing activity and safety of each therapeutic agent across the patient treatment journey.

TEAEs reported by patients in both BRAF status groups treated with cabozantinib were comparable, manageable, and consistent with observations in the full safety population of COSMIC-311 (6, 9), in which the most common grade 3/4 TEAEs were palmar-plantar erythrodysesthesia, hypertension, fatigue, diarrhea, and hypocalcemia. Although over half of patients required dose reductions due to adverse events (AEs), <10% discontinued treatment due to AEs unrelated to DTC, supporting the appropriate management of TEAEs is through proactive AE management and dose modifications (6, 9).

Limitations of this subgroup analysis include its exploratory nature and a relatively small subgroup size that may introduce biases. The results are to be considered hypothesis-generating because subgroups were not powered to show differences between treatment arms. Our observations nevertheless may have clinical implications for cabozantinib treatment of RAIR-DTC, in which *BRAF*^V600E^ is prevalent.

In conclusion, this subgroup analysis of the COSMIC-311 phase 3 trial showed that treatment with cabozantinib improved clinical outcomes, including a prolonged PFS and higher ORR, compared with placebo in patients with either *BRAF*^wt^ or *BRAF*^V600E^. The magnitude of clinical outcomes was similar to that seen in the intention-to-treat population regardless of the *BRAF* mutational status. The observed efficacy along with a manageable safety profile further supports cabozantinib as an efficacious treatment following VEGFR-targeted therapy in patients with RAIR-DTC including those with *BRAF*^V600E^ mutations.

## Data Availability

Study-level clinical data from this study may be be made available upon reasonable request from a qualified medical or scientific professional for the specific purpose laid out in that request and may include deidentified individual participant data. Requests to access the datasets should be directed to publications@exelixis.com. The data for this request will be available after a data access agreement has been signed.
